# Discriminative Long-Distance Transport of Selenate and Selenite Triggers Glutathione Oxidation in Specific Subcellular Compartments of Root and Shoot Cells in *Arabidopsis*

**DOI:** 10.3389/fpls.2022.894479

**Published:** 2022-06-24

**Authors:** Muhammad Sayyar Khan, Anna Soyk, Ingo Wolf, Miriam Peter, Andreas J. Meyer, Thomas Rausch, Markus Wirtz, Rüdiger Hell

**Affiliations:** ^1^Centre for Organismal Studies, Heidelberg University, Heidelberg, Germany; ^2^Institute of Biotechnology and Genetic Engineering, The University of Agriculture, Peshawar, Pakistan; ^3^INRES - Chemical Signalling, University of Bonn, Bonn, Germany

**Keywords:** selenium, toxicity, oxidation, compartmentation, roGFP2

## Abstract

Selenium is an essential trace element required for seleno-protein synthesis in many eukaryotic cells excluding higher plants. However, a substantial fraction of organically bound selenide in human nutrition is directly or indirectly derived from plants, which assimilate inorganic selenium into organic seleno-compounds. In humans, selenium deficiency is associated with several health disorders Despite its importance for human health, selenium assimilation and metabolism is barely understood in plants. Here, we analyzed the impact of the two dominant forms of soil-available selenium, selenite and selenate, on plant development and selenium partitioning in plants. We found that the reference plant *Arabidopsis thaliana* discriminated between selenate and selenite application. In contrast to selenite, selenate was predominantly deposited in leaves. This explicit deposition of selenate caused chlorosis and impaired plant morphology, which was not observed upon selenite application. However, only selenate triggered the accumulation of the macronutrient sulfur, the sister element of selenium in the oxygen group. To understand the oxidation state-specific toxicity mechanisms for selenium in plants, we quantified the impact of selenate and selenite on the redox environment in the plastids and the cytosol in a time-resolved manner. Surprisingly, we found that selenite first caused the oxidation of the plastid-localized glutathione pool and had a marginal impact on the redox state of the cytosolic glutathione pool, specifically in roots. In contrast, selenate application caused more vigorous oxidation of the cytosolic glutathione pool but also impaired the plastidic redox environment. In agreement with the predominant deposition in leaves, the selenate-induced oxidation of both glutathione pools was more pronounced in leaves than in roots. Our results demonstrate that Se-species dependent differences in Se partitioning substantially contribute to whole plant Se toxicity and that these Se species have subcellular compartment-specific impacts on the glutathione redox buffer that correlate with toxicity symptoms.

## Introduction

The element selenium (Se) is essential for the synthesis of seleno-proteins in prokaryotic and eukaryotic species from diverse phyla (Stadtman, 1990; Terry et al., 2000). In algae and animals, Se-containing cysteine (Se-Cys) is specifically incorporated into seleno-proteins during translation *via* a Se-Cys selective t-RNA (Novoselov et al., 2002). In mammals, 25 seleno-proteins are known many of which have crucial functions, especially in stress responses (Lu and Holmgren, 2009). Hence, the health-promoting properties of Se have been highlighted in numerous studies (Finley et al., 2001; Tsuji et al., 2021). Se deficiency causes hypothyroidism and increases the risk for diseases of the cardiovascular, osseus and nervous systems (Rayman, 2000).

Albeit Se is required only in trace amounts, a substantial number of people are suffering from Se deficient diet (Combs, 2001). This is because the ultimate source of Se in the diet are higher plants, which adsorb Se from the soils, being the sister element of sulfur in the chalcogen group of the periodic table. Particularly, in several European countries and certain provinces of China, soils exhibit a low Se content, causing significant Se malnutrition in the local populations [reviewed in Zhu et al. (2009); Gupta and Gupta (2017)]. Global warming will reinforce Se deficiency due to enhanced loss of Se from cropland and the decreased uptake of Se from the soil during water limitation, which will worsen Se nutrition of future generations (Ahmad et al., 2016; Jones et al., 2017).

For higher plants Se is considered as beneficial but not an essential nutrient [noteworthy plants do not possess seleno-proteins (Novoselov et al., 2002; Navrot et al., 2006)] and the dynamic range between deficiency and toxicity of Se is very narrow (Rayman, 2000; Zhu et al., 2009). Exceptions of this rule are Se hyperaccumulating plant species, which store substantial amounts of Se in the non-proteinogenic amino acid methyl-Se-Cys [up to 1% of the plant dry weight; (Neuhierl and Boeck, 1996; Van Hoewyk, 2013; Pilon-Smits, 2019)], demonstrating that plants have the genetic capability to serve as a valuable Se dietary source. To explore and subsequently exploit the full capability of crops as Se sources, a thorough understanding of the molecular mechanisms determining Se toxicity and Se metabolization in plants is required (Schiavon and Pilon-Smits, 2017b).

Selenate and selenite are the two major forms of Se available for plant uptake from the soil and give rise to 95% of Se toxicity in human cells (Gebreeyessus and Zewge, 2018). The distribution of both forms in the soil water depends on the redox potential, pH and aeration of the pedosphere (Schiavon and Pilon-Smits, 2017a). In well-aerated soils, selenate is the most oxidized and bioavailable form of Se (Van Hoewyk, 2013). Because of its chemical similarity to sulfate, selenate can be taken up *via* sulfate transporters (Terry et al., 2000; El Kassis et al., 2007; El Mehdawi et al., 2018). The selenite uptake system of plants is less characterized. Selenite absorption was supposed to occur passively *via* pores or channels (Terry et al., 2000; Zhao et al., 2010) or might be actively facilitated by plasma membrane resident transporters (Shrift and Ulrich, 1969; Arvy, 1993), of which phosphate transporters appear to be the best candidates (Li et al., 2008; Zhang et al., 2014). Long-distance transport of Se from the roots to the shoots has been reported. Several studies suggested a more rapid transport mechanism for selenate than selenite (Arvy, 1993; Li et al., 2008), albeit also comparable uptake kinetics for selenate and selenite into roots and shoots have been reported (Zhang et al., 2003).

Since selenate and selenite can be incorporated into Se-Cys in plants *via* the assimilatory sulfate reduction pathway (Sors et al., 2005; Takahashi et al., 2011), Se toxicity is at least partly caused by misincorporation of Se-Cys into proteins (reviewed in Schiavon and Pilon-Smits, 2017a). This will result in misfolding of proteins or misassembly of multi-protein complexes due to the substantially different redox properties of Se-Cys and cysteine affecting disulfide-bridge formation essential for tertiary and quaternary structure formation. Consequently, Se treatment induces the ubiquitin-proteasome system, which degrades misfolded and/or orphaned proteins in the nucleus and the cytosol (Vierstra, 2009), and proteasome depleted Arabidopsis mutants are more sensitive to Se-treatment (Sabbagh and Van Hoewyk, 2012). Furthermore, over-expression of a Se-Cys degrading seleno-cysteine lyase confers higher tolerance to Se (Van Hoewyk et al., 2007). Finally, the evolutionary selected strategy in Se hyperaccumulators is the storage of reduced Se in a non-proteinogenic amino acid (i.e., methyl-seleno-cysteine), further emphasizing the efficient assimilatory reduction capacity of these plants for Se (Neuhierl and Boeck, 1996; LeDuc et al., 2004).

Recent studies have highlighted the capacity of selenate and selenite to induce reactive oxygen species (ROS) accumulation, which was proposed to contribute to Se toxicity in plants (Freeman et al., 2010; Grant et al., 2011). The thiol group of Cys is critical for coordinating Fe/S clusters in numerous electron transfer reaction centers of proteins (Roland et al., 2020). Consequently, unwanted replacement of Cys with Se-Cys might interfere substantially with efficient electron transfer within the electron transport chains operating in photosynthesis and respiration (Van Hoewyk, 2013) The thiol group of Cys is critical for coordinating Fe/S clusters in numerous electron transfer reaction centers of proteins (Roland et al., 2020). Consequently, unwanted replacement of Cys with Se-Cys might interfere substantially with efficient electron transfer within the electron transport chains operating in photosynthesis and respiration (Van Hoewyk, 2013). Inefficient electron transfer in these chains will result in electron spillover and consequently ROS formation in plastids and/or mitochondria. Thus, the Se-triggered accumulation of ROS might also occur as a consequence of unwanted incorporation of Se-Cys into proteins. Another potential explanation for the Se-induced ROS accumulation might be the replacement of Cys with Se-Cys in the redox buffer glutathione. Both explanations require assimilatory reduction of selenate or selenite into Se-Cys.

In this study, we tested Se toxicity in shoots and roots induced by feeding of selenate or selenite *via* the roots. We assessed the consequences of Se species for redox homeostasis by application of the genetically encoded redox-sensor ro-GFP2, allowing us to dissect the sub-cellular compartment-specific impact of selenate or selenite on the redox milieus in both plant organs.

## Materials and Methods

### Plant Material and Growth Conditions

All work was performed with *Arabidopsis thaliana* ecotype Columbia-0 (Col-0). For the analysis of the cytosolic and the plastidic glutathione redox potential, wild-type plants expressing the Grx1-roGFP2 sensor in one of the analyzed subcellular compartments were applied. These transgenic lines have been previously established and extensively characterized (Meyer et al., 2007; Schwarzländer et al., 2008).

#### Growth on Sterile Medium

For growth on sterile media, seeds were surface-sterilized with 70% (v/v) ethanol (5 min) and 6% (v/v) NaClO (2 min) followed by three washing steps with sterile water. After 2 days of stratification at 4°C, the seeds were germinated under short-day conditions on a solid medium. In all cases, plants were kept in the climate chamber under short-day conditions (8.5 h light). The light intensity in the growth chamber was set to 100 μEm^−2^ s^−1^, whereas the relative humidity was kept at 50%. The temperatures during the day and night were set at 22 and 18°C, respectively, as established in Forieri et al. (2017).

The comparison of concentration-dependent toxicity triggered by selenate or selenite was performed with six-day-old seedlings grown on solidified Arabidopsis (*At*-medium) medium (Haughn and Somerville, 1986). These seedlings were challenged for 15 days with varying concentrations of up to 200 μM selenate or selenite.

To analyze the organ-specific impact of selenate and selenite on the deposition of selenium, the assimilatory sulfate reduction pathway, and the intracellular glutathione redox potential, wild type or wild type expressing the Grx1-roGFP2 sensors were grown in hydroponic culture in the presence or absence of both tested selenium species. For hydroponic cultures, seeds were germinated in Eppendorf tubes placed in small boxes (0.25 L) as described by Tocquin et al. (2003), containing half-strength Hoagland solution [2.5 mMCa(NO_3_)_2_, 2.5 mM KNO_3_, 0.5 mM MgSO_4_, 0.5 mM KH_2_PO_4_, 40 μM Fe-EDTA, 25 mMH_3_BO_3_, 2.25 μMnCl_2_, 1.9 mMZnSO_4_, 0.15 μMCuSO_4_, and 0.05 μM(NH_4_)_6_Mo_7_O_24_, pH 5.8 to 6.0]. The media were exchanged every 7 days until the age of 6 weeks. At this stage, plants were transferred to a medium containing either 50 μM selenite (Na_2_SeO_3_) or selenate (Na_2_SeO_4_), and grown for an additional week prior to metabolite analysis. For *in vivo* imaging, the plants were supplemented for 3–120 h with 50 μM selenite (Na_2_SeO_3_) or selenate (Na_2_SeO_4_), respectively. Control plants were kept under the same conditions as described above.

### Determination of Metabolites and Elemental Analysis

Hydrophilic metabolites from the leaves and roots of 7-week-old hydroponically grown Col-0 plants were extracted according to Wirtz and Hell (2007). Thiol and OAS were quantified after derivatization with monobromobimane (Calbiochem, EMD Chemicals) and AccQ-Tag reagent (Waters), respectively. The derivatization procedure and separation of thiol derivatives were performed as described by Wirtz et al. (2004), using the same HPLC system. Separation and quantification of anions were carried out according to (Wirtz and Hell, 2007) using a 10-fold diluted extract in water.

For the determination of total sulfur, Se and phosphorous, tissue samples of the 7 weeks old hydroponically-grown Col-0 plants were dried in an oven for 3 days. Dried samples (approx. 10 mg) were digested in 2.5 ml 65% HNO_3_. The samples were allowed to stand at room temperature for 2 days under the fume hood and then heated for 1 h at 95°C in a heating block. Afterward, samples were further heated for an additional 3 h at 105°C. Samples were mixed occasionally, and the contents of the tubes were monitored after short intervals. In case of any reduction to less than 0.5 ml, 0.5 to 2 ml HNO_3_ was added to each tube after cooling down the tubes. In the end, the contents of each tube were filled to a total of 10 ml with ultrapure water and closed with a lid. Total element contents were determined by ICP-AES (Thermo Elemental, Dreieich, Germany) using an IRIS Advantage Duo ER/S as described in (Haydon et al., 2012).

### *In vitro* Analysis of Selenium Species-Induced Glutathione Oxidation

The capacity of selenium species to oxidize glutathione was determined based on the reduction of the glutathione disulfide by Arabidopsis glutathione disulfide reductase 1 (GR1) in the presence of NADPH (Marty et al., 2019). The assay was performed in a total volume of 200 μl in transparent 96-well plates in a Fluostar Optima plate reader (BMG, Offenburg). Protein and substrates were added in the following concentrations: 2 mM GSH, 100 μM NADPH, 0.5 U GR1, 10–100 μM Na_2_SeO_3_, filled up with 100 mM K_2_HPO_4_/KH_2_PO_4_ pH 7.2, 1 mM EDTA. GSH and NADPH were always freshly prepared. The reaction was initiated by injection of Na_2_SeO_3_, and the activity was monitored as absorbance of NADPH at 340 nm for 6 min.

### *In vivo* roGFP2 Imaging

Ratiometric redox imaging of the fluorescence sensor roGFP2 *in vivo* was performed with an inverted Zeiss confocal laser scan microscope LSM 510 META. Imaging was conducted using a 25x lens (Zeiss 25× 0.8 N.A. Plan-NEOFLUAR multi-immersion lens) and a numerical zoom of 3x. Images were taken from lower epidermal leaf cells and the root elongation zone. Images were taken in multitrack mode with line switching between a diode laser for excitation at 405 nm and an argon laser for excitation at 488 nm. roGFP2 fluorescence was collected with a bandpass filter of 505–530 nm as previously established in Speiser et al. (2018). Ratiometric image analysis was performed using a custom Matlab analysis suite (Schwarzländer et al., 2008; Fricker, 2016). Pixels with intensities within 10% saturation or with less than 2 standard deviation units above background were ignored for the analysis.

### Basic Statistical Analysis

Statistical analysis was conducted using SigmaPlot 12.0. Means from different sets of data were analyzed for statistically significant differences with the Holm-Sidak One-Way ANOVA test or the student’s t-test. Significant differences (*p* < 0.05) are indicated with different letters or asterisks, respectively.

## Results

### Only Selenate Impairs Shoot Growth, Albeit Both Selenate and Selenite Are Toxic for Root Cells

**Figure 1:**
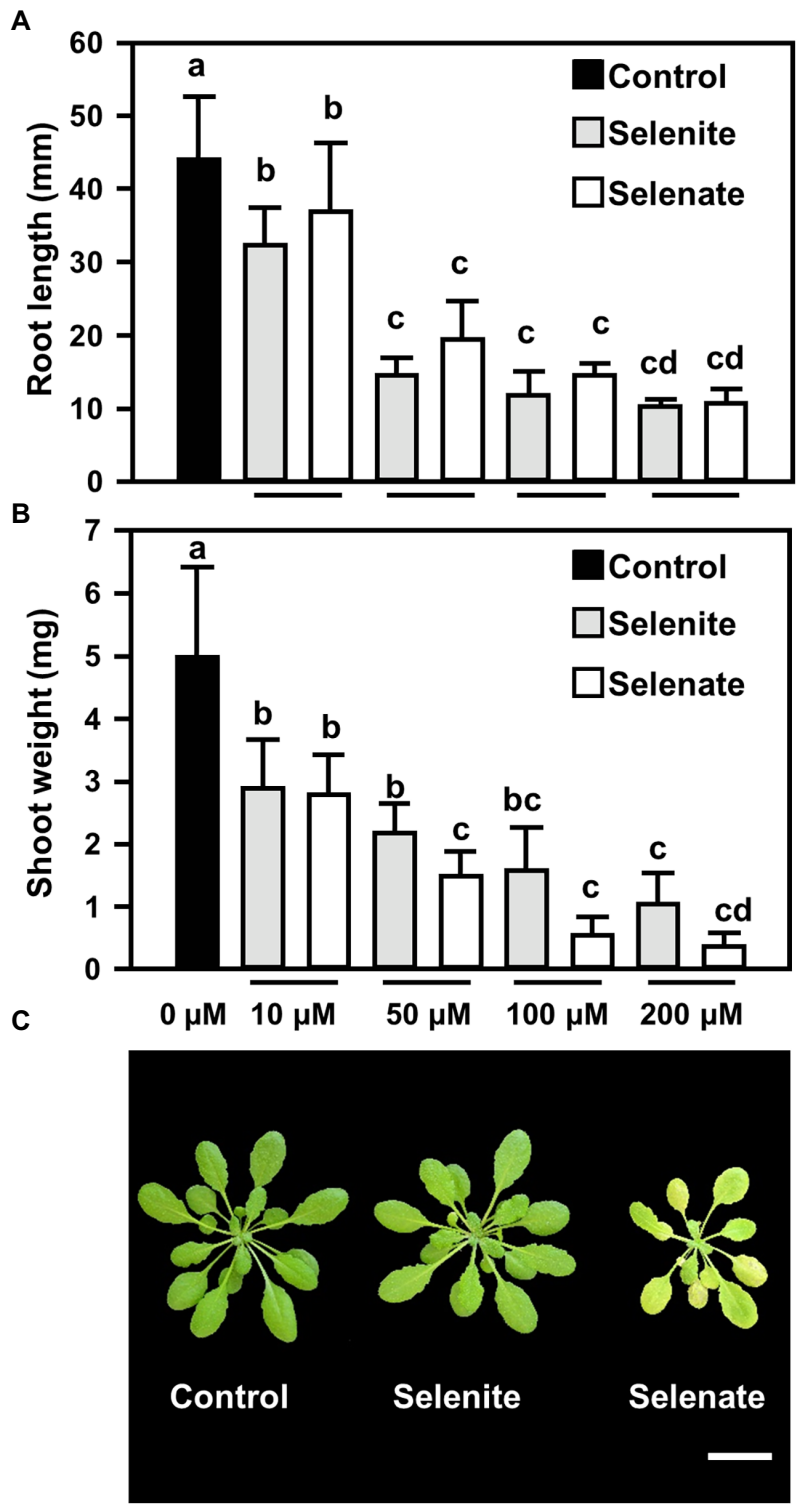
Impact of selenite and selenate treatments on the roots and leaves of Arabidopsis plants **(A,B)** Impact of increasing concentrations of selenite and selenate (0–200 μM) on root length **(A)** and shoots fresh weight **(B)** of *Arabidopsis*. Six-days-old seedlings were challenged for additional 15 days on At-medium (Control, black bars) or At-medium containing either selenite (gray bars) or selenate (white bars). Different lowercase letters indicate individual groups identified by pairwise multiple comparisons with a Holm-Sidak one-way ANOVA (*p* = 0.05, n = 7). **(C)** Top view of six-week-old hydroponically grown Arabidopsis plants that were either challenged for seven days with ½ Hoagland media supplemented with selenite (50 μM Na_2_SeO_3_) or selenate (50 μM Na_2_SeO_4_). The control plants were grown under the same conditions (100 μE light for 8 h, 50% humidity and 22°C day/18°C night) on ½ Hoagland medium lacking selenium. Images were digitally extracted for comparison. Scale bar = 1 cm. FW, fresh weight.

Se toxicity induced by selenate (Na_2_SeO_4_) or selenite (Na_2_SeO_3_) was assessed with 6 days old *A. thaliana* wild-type seedlings that were transferred to solidified medium supplemented with increasing concentrations of either selenite or selenate (0 to 200 μM) and grown for further 15 days. The impact of both Se species on root growth was comparable and clearly detectable after applying 10 μM Se (Figure 1A). This low Se application also caused a significant decrease of shoot growth, which was independent of the Se oxidation state at 10 μM Se. At higher Se concentrations, selenate had a stronger impact on shoot growth than selenite application (Figure 1B). To independently confirm this significantly stronger impact of selenate on shoot growth, we applied an intermediate concentration (50 μM) of both Se species to six-week-old hydroponically grown plants. After Se application for 7 days, the leaves of the selenate-treated plants were more chlorotic than the control and selenite-treated plants (Figure 1C). Taken together, these data demonstrated that selenate had a significantly more pronounced impact on shoot growth than selenite.

### Preferentially Selenate Is Transported to the Shoots, While Selenite Remains in the Roots

**Figure 2:**
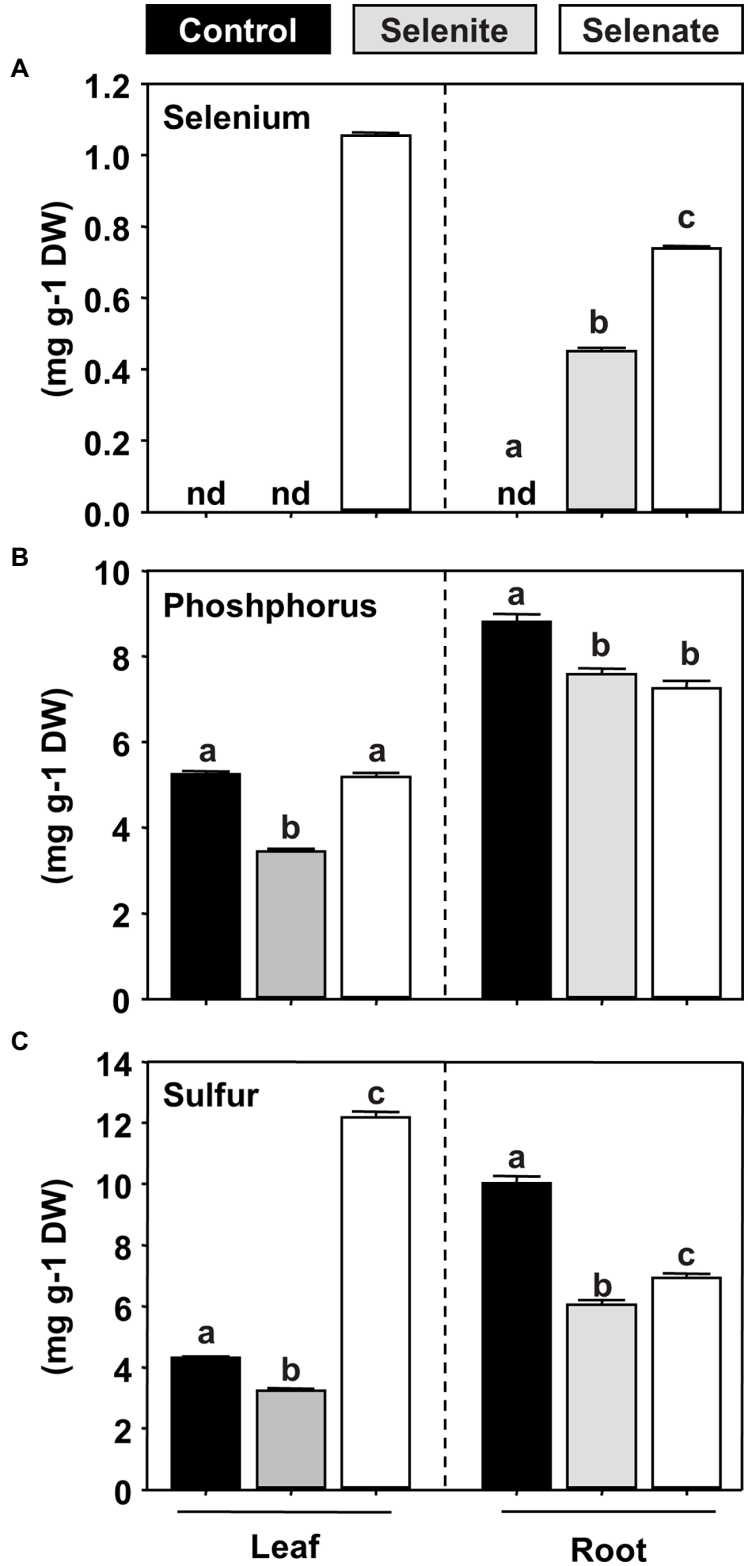
Impact of selenite and selenate application on the partitioning of selenium, phosphorus, and sulfur in Arabidopsis. **(A–C)** Deposition of selenium **(A)**, phosphorus **(B)**, and sulfur **(C)** in leaves and roots of seven-week-old hydroponically grown plants challenged for one week with 50 μM selenite (gray), 50 μM selenate (white) or no additional selenium (Control, black). Different lowercase letters indicate individual groups identified by pairwise multiple comparisons with a Holm-Sidak one-way ANOVA (*p* = 0.05, n = 3). n.d., not detectable, DW, dry weight.

The shoot-specific phenotype of selenate-treated *Arabidopsis* prompted us to investigate the Se partitioning in selenate and selenite-treated hydroponically grown plants. As expected, Se was undetectable in the non-treated control plants. However, the application of both Se species resulted in a substantial accumulation of Se in the roots, while only selenate treatment resulted in detectable Se translocation to the shoot (Figure 2A). Thus, the shoot-specific impact of selenate could be explained by the oxidation state-specific transport of Se to this organ in Arabidopsis. Since selenite and selenate are supposed to be transported by either the phosphate translocation or the sulfate translocation systems, respectively, we also analyzed total phosphorus and sulfur contents in both organs. In agreement with the hypothesis of membrane transport of selenite by phosphate transporters, we found a specific decrease of phosphorus contents in shoots of selenite-treated plants, while selenate treatment had no impact on shoot phosphorus deposition (Figure 2B). Treatment with both Se species resulted in a similarly marginal decrease of phosphorus contents in roots, which might be attributed to general Se toxicity in this organ. In addition, sulfur contents decreased in the roots of Se-treated plants, and this decrease was independent of the Se oxidation state (Figure 2C). Surprisingly, we found a 3-fold accumulation of sulfur in the shoots of selenate-treated plants, underpinning the organ-specific impact of selenate on shoot metabolism. In contrast, selenite application decreased sulfur deposition in the shoot, albeit this decrease was moderate (Figure 2C).

### Selenate Exposure Perturbs the Assimilatory Sulfate Reduction Pathway

**Figure 3:**
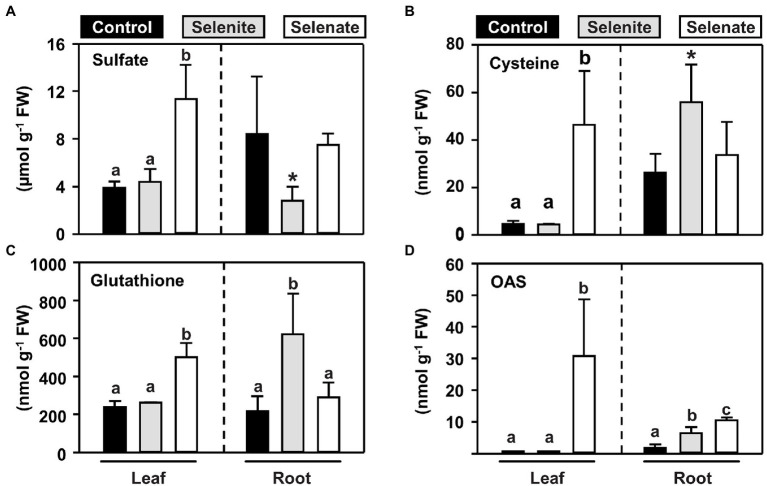
Organ-specific impact of selenite and selenate application on key metabolites of the reductive sulfur assimilation pathways. **(A–D)** Steady-state levels of sulfate **(A)**, cysteine **(B)**, glutathione **(C)** and the carbon-nitrogen backbone for sulfide/selenide incorporation, OAS **(D)** in leaves and roots of seven-weeks-old hydroponically grown Arabidopsis plants challenged for one week with 50 μM selenite (gray), 50 μM selenate (white) or no additional selenium (control, black). Different lowercase letters indicate individual groups identified by pairwise multiple comparisons with a Holm-Sidak one-way ANOVA (P, 0.05, n = 7). If the power of the α-test was too low in the one-way ANOVA, we tested the statistical difference between control and the single selenium treatment by a paired students t-test (^*^p = <0.05). FW, fresh weight.

Due to the similar chemical properties, Se is believed to share the initial route for its uptake and reductive assimilation with sulfur (Terry et al., 2000). However, when broccoli, a crop belonging to the family of *Brassicaceae* like Arabidopsis, was fertilized with non-toxic concentrations of selenate, foliar sulfur contents increased, which was predominantly caused by sulfate accumulation and not by reduced sulfur-containing compounds (Hsu et al., 2011). To understand the impact of Se species differing in their oxidation state on plant sulfur metabolism at the organismal level, we determined the steady-state levels of primary sulfur metabolites in the leaves and roots of selenate and selenite treated plants. The most abundant sulfur-containing low molecular-weight compound is sulfate, which can be stored in the vacuole of plants. Selenate feeding *via* the roots triggered sulfate accumulation specifically in the shoots, while the steady-state sulfate level in the root remained unaffected (Figure 3A). Remarkably, the steady-state levels of the reduced sulfur-containing thiols cysteine and glutathione mirrored the selenate-induced changes of steady-state sulfate levels in shoots and roots (Figures 3B,C).

In contrast to selenate, the application of selenite did not trigger an accumulation of sulfate or thiols in the shoots, which is consistent with its sole deposition in the root. In this organ, selenite application resulted in lowered sulfate levels, but at the same time, thiols accumulated, suggesting that selenite triggered the assimilatory sulfate reduction in roots. In support of this hypothesis, *O*-acetylserine (OAS), the carbon and nitrogen-containing precursor for sulfide incorporation into cysteine, also accumulated in the root but not in the shoot (Figure 3D). Albeit OAS does not contain sulfur, it is dedicated to sulfur-metabolism and serves as a trigger for transcriptional induction of sulfate transporters (Smith et al., 1997; reviewed in Takahashi et al., 2011). Remarkably, only selenate caused a 30-fold accumulation of foliar OAS levels, while selenite had no impact on OAS steady-state levels in leaves (Figure 3D). In the roots, selenate also caused OAS accumulation, albeit the amplitude was lower when compared to shoot. These findings strongly suggested that shoot-specific selenate toxicity was caused by perturbation of reductive sulfate assimilation, which activates sulfate/selenate transporter capacities *via* accumulation of OAS in roots and shoots, leading to shoot-specific deposition of Se upon selenate treatment.

### *In vitro* Selenite Can Be Reduced to Selenide at the Expense of Glutathione

Since selenite had a significantly different impact on sulfur metabolism and plant growth compared to selenate, we aimed next to understand how selenate and selenite are assimilated into organic Se compounds. The main sink of reduced Se is Se-Cys, which might be directly incorporated into proteins or serve as a Se donor for Se-Met or Se-GSH production. Since selenide is the precursor for Se-Cys, we addressed the reduction of both Se species to selenide.

**Figure 4:**
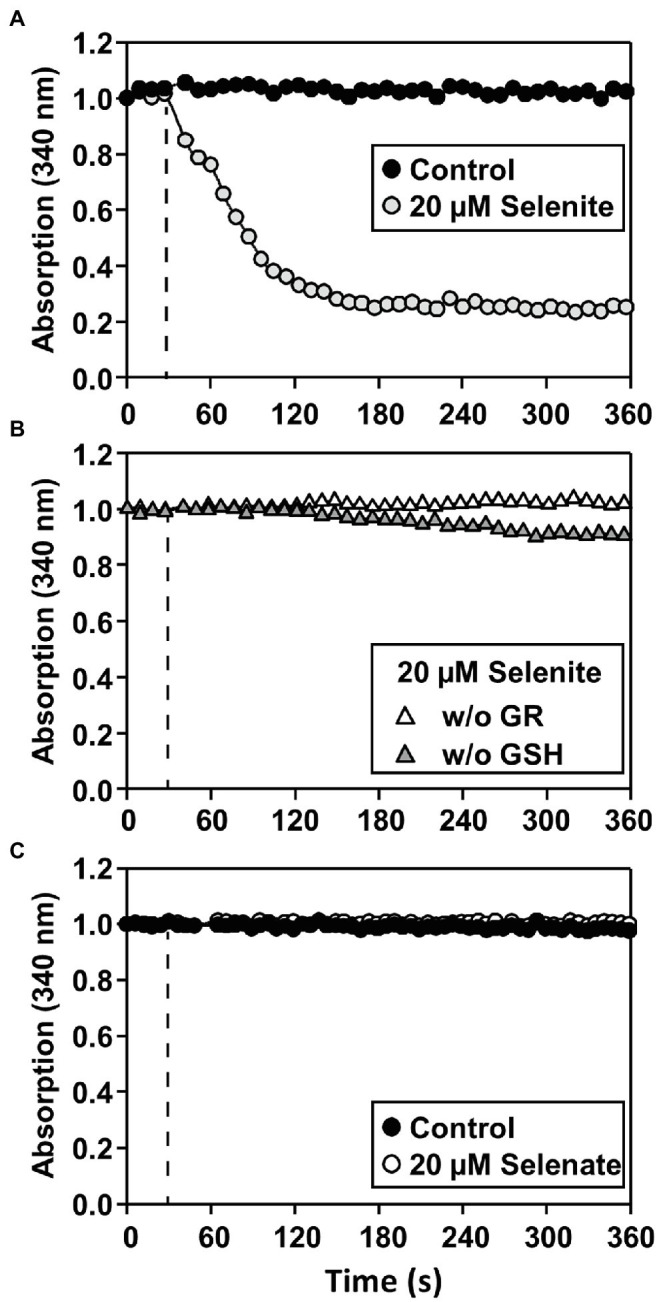
Only selenite can be reduced by GSH in a cell-free system. **(A–C)** *In vitro* assay for determining the reduction of selenite **(A,B)** or selenate **(C)** by glutathione resulting in glutathione disulfide (GSSG). The assay allows quantification of selenium reduction by coupling the reduction of the byproduct GSSG to GSH by glutathione disulfide reductase (GR) under consumption of NADPH, which can be quantified at 340 nm. **(A)** The assay (0.5 μM GR, 2 mM GSH, 100 μM NADPH) was preincubated at the reaction temperature, and the reaction was started (dashed line) by the addition of 20 μM selenite (gray circles) or water (Control, black circles). **(B)** As controls for the specificity of the NADPH-dependent reduction of GSSG by GR, GSH (white triangles) or GR (gray triangles) was omitted from the assay. In contrast to selenite application, **(C)** application of selenate (white circles) did not result in GSSG formation and was indistinguishable from control (black circles). The selenate assay was performed under the identical conditions described in **(A)** for selenite. All assays were repeated in triplicates at two individual time points and showed similar results.

It has been previously postulated that the cytotoxicity of selenite is potentially a consequence of the oxidation of glutathione during selenite reduction (Wallenberg et al., 2010). To test the hypothesis of non-enzymatic selenite reduction at the expense of reduced glutathione (GSH), an enzymatic assay based on NADPH-dependent glutathione disulfide reductase (GR) was developed. To this end Arabidopsis GR1 protein was recombinantly expressed and purified. Selenite reduction was monitored *via* the generation of glutathione disulfide (GSSG) from GSH. The GSSG was recycled to GSH by GR under the consumption of NADPH, which was recorded by monitoring the absorbance change at 340 nm. After selenite injection a rapid decrease of NADPH absorption was observed, indicating the reduction of generated GSSG by GR activity (Figure 4A). This rapid decrease was dependent on the presence of both GSH and GR (Figure 4B), demonstrating that the monitored NADPH consumption is likely caused by non-enzymatic reduction of selenite to selenide with electrons derived from GSH leading to the concomitant formation of GSSG. As expected by their redox potentials, GSH failed to reduce selenate efficiently to selenite/selenide (Figure 4C). These findings demonstrated that selenite might be able to oxidize the cellular glutathione pool *in vivo* and thus enhance its cytotoxicity. Remarkably, while selenate was found to be more toxic *in vivo*, it was not causing any oxidation of glutathione in a cell-free system.

### Selenate Caused Significantly Stronger Oxidation of the Cytosolic Redox Milieu in Roots Than Selenite

**Figure 5:**
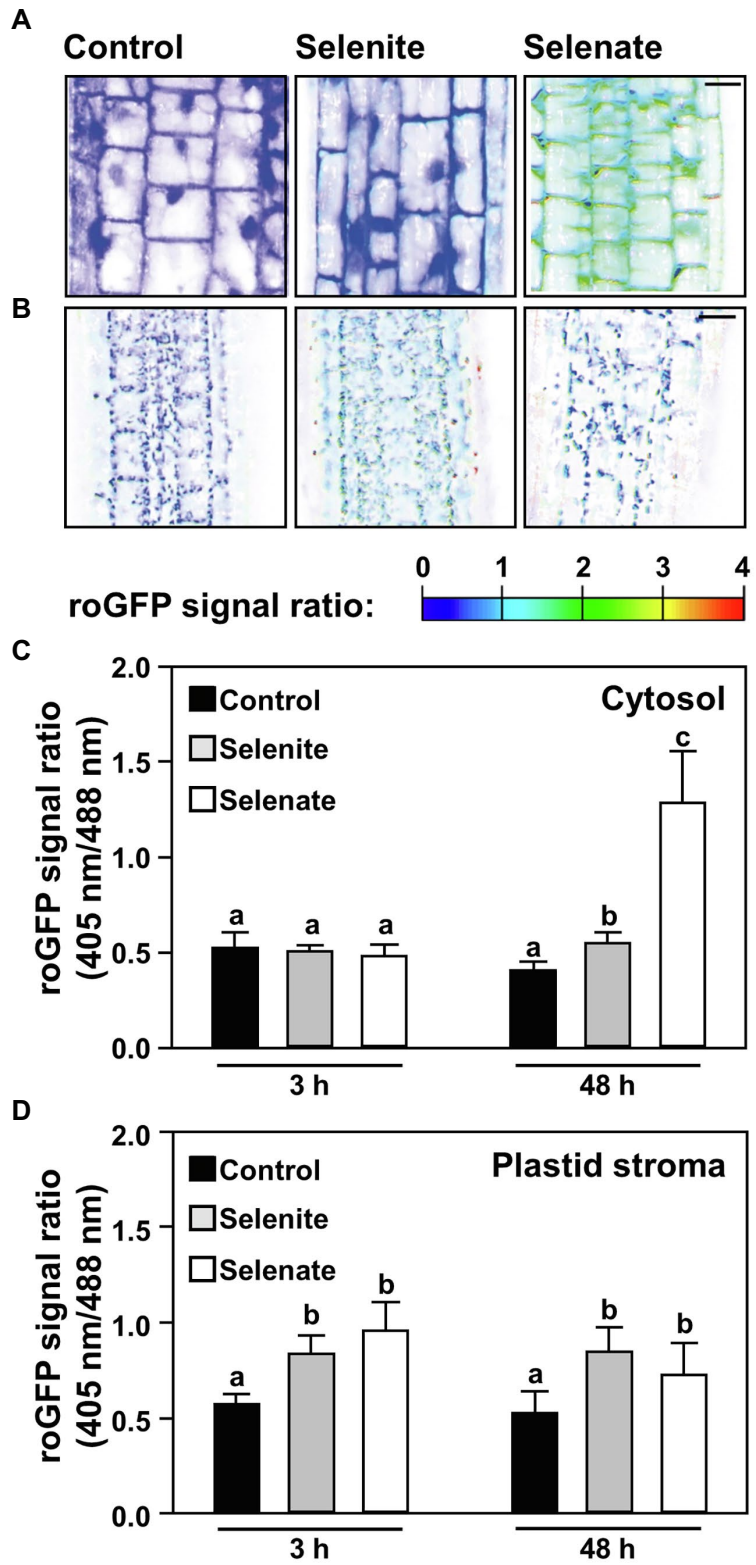
Time-resolved impact of selenium species on the cytosolic and the plastid glutathione redox state in roots of Arabidopsis. **(A,B)** Representative false-color images of the Grx1-roGFP2 signal ratio in the cytosol **(A)** or plastids **(B)** of roots treated with selenite, selenate or without additional selenium supply for indicated time points. Arabidopsis plants expressing the Grx1-roGFP2 sensor in the cytosol or plastids were treated for up to 2 days in ½ Hoagland medium (control), supplemented with either 50 μM Na_2_SeO_3_ (selenite) or 50 μM Na_2_SeO_4_ (selenate). roGFP2 was excited at 405 nm and 488 nm, respectively, and fluorescence was collected at 505–530 nm. The 405/488-nm fluorescence ratio is shown on a false-color scale spanning full reduction of roGFP2 (blue) to full oxidation (red). Images were digitally enhanced for comparison. Scale bar = 20 μm. **(C,D)** The roGFP2 fluorescence ratio is indicating relative changes in the glutathione redox potential in the cytosol **(C)** or the plastid stroma **(D)** of roots treated for indicated times with ½ Hoagland medium (control, black bars), supplemented with selenite (gray bars) or selenate (white bars). Data are shown as mean values ± SD. Different lowercase letters indicate individual groups identified by pairwise multiple comparisons with a Holm-Sidak one-way ANOVA (*p* = 0.05, n = 5–15).

Next, we addressed the impact of both Se species on the glutathione redox potential in the cytosol of root cells. To this end, we applied selenate and selenite for up to 48 h and analyzed the impact of both Se species in a time-resolved manner by non-invasive live-cell imaging in Arabidopsis wild type expressing the Grx1-roGFP2 sensor in the cytosol (Meyer et al., 2007; Gutscher et al., 2008). Short-term application (3 h) of selenite or selenate did not significantly oxidize the cytosolic glutathione pool as indicated by the unaffected 405/488-nm roGFP2 emission signal ratio obtained after sequential excitation of roGFP2 at 405 nm and 488 nm. More prolonged selenate application (48 h) caused significant oxidation of roGFP2, which strongly suggests oxidation of the cytosolic glutathione pool. The selenate impact was significantly higher than the only marginal oxidizing impact of selenite in the cytosol (Figures 5A,C). Based on the more substantial impact of selenate on glutathione oxidation compared to selenite and the results of Se-induced glutathione oxidation in the cell-free system (Figures 4A,C), we hypothesized that the *in vivo* oxidation of the cytosolic glutathione pool is not caused directly by selenate. To address if selenate might be first reduced to selenite or selenide to trigger glutathione oxidation, we tested the impact of both Se species on the glutathione pool in the plastids of root cells.

### Selenate and Selenite Oxidize the Plastid Glutathione Pool Prior to Affecting the Cytosolic Glutathione Pool in Roots

The plastid glutathione pool of roots cells was substantially oxidized after short-term application of selenate but was not oxidized further upon prolonged treatment. Thus, selenate application oxidized first the plastid glutathione pool, albeit the external feeding of selenate implies that the selenate passed the cytosol of root cells before it was taken up into plastids. Also, selenite application quickly oxidized the glutathione pool of plastids in root cells (Figure 5D) but did not affect the cytosolic glutathione pool (Figure 5C).

If the significantly stronger oxidation of the cytosolic glutathione pool by selenate as compared to selenite (Figures 5A, C) is caused by the 1.8-fold higher Se accumulation in roots upon selenate feeding, or a different subcellular localization of both Se species triggered by discriminative transport within root cells (as suggested by results shown Figure 2) is currently unclear.

### Only Selenate Oxidizes the Cytosolic and Plastid Glutathione Pool in Leaves

**Figure 6:**
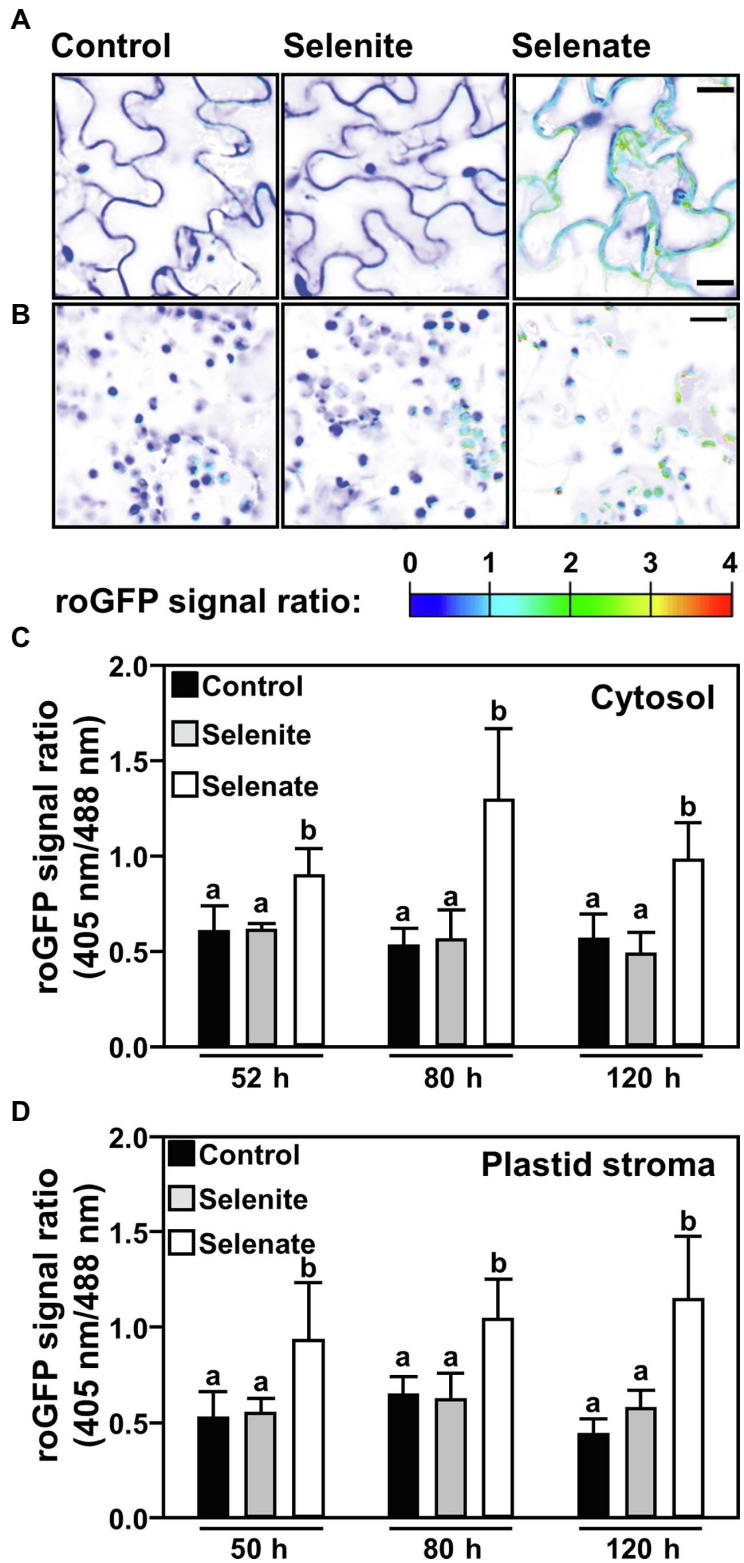
Time-resolved impact of selenium species on the cytosolic and the plastid glutathione redox state in leaves of *Arabidopsis*. **(A,B)** Representative false-color images of the Grx1-roGFP2 signal ratio in the cytosol **(A)** or plastids **(B)** of leaves from hydroponically grown plants treated with selenite, selenate or without additional selenium supply for indicated time points *via* the root system. Arabidopsis plants expressing the Grx1-roGFP2 sensor in the cytosol or plastids were treated for up to 2 days in ½ Hoagland medium (control), supplemented with either 50 μM Na_2_SeO_3_ (selenite) or 50 μM Na_2_SeO_4_ (selenate). roGFP2 was excited at 405 nm and 488 nm, respectively, and fluorescence was collected at 505–530 nm. The 405/488-nm fluorescence ratio is shown on a false-color scale spanning full reduction of roGFP2 (blue) to full oxidation (red). Images were digitally extracted for comparison. Scale bar = 20 μm. **(C,D)** The roGFP2 ratio signal is indicating relative changes of the glutathione redox potential in the cytosol **(C)** or the plastid stroma **(D)** of leaves treated for the indicated times with ½ Hoagland medium (control, black), supplemented with selenite (gray) or selenate (white). Data are shown as mean values ± SD. Different lowercase letters indicate individual groups identified by pairwise multiple comparisons with a Holm-Sidak one-way ANOVA (*p* = 0.05, n = 8–20).

Since only selenate feeding *via* the roots caused substantial chlorosis in Arabidopsis leaves, we tested if the Se triggered perturbation of the plastid redox potential might be the cause. Indeed, we found that feeding of selenate oxidized the cellular glutathione pool and preceded visible chlorosis of leaves. Already 2 days after the transfer to selenate-containing medium the cytosolic and plastid glutathione pools were highly oxidized; this degree of oxidation remained high upon continuous selenate feeding (Figure 6). As expected, based on its sole deposition in roots, selenite feeding did not impair the redox milieu in leaf cells of selenite-treated plants (Figure 6). These findings demonstrate that the specific impact of Se species on plant growth correlates with the discriminative transport of selenite and selenate, resulting in pronounced oxidation of the cytosolic and plastidic glutathione pools in leaf cells.

## Discussion

This study identifies the selective transport of selenate compared to selenite as pivotal for Se toxicity in *Arabidopsis*. The basis for the distinct consequences of Se species is the operation of a specific transport systems once inside the plant body distinguishing between selenate and selenite. Previous studies have already suggested the phosphate uptake system as the prime candidate for selenite uptake into roots of monocotyledonous plants (Li et al., 2008; Zhang et al., 2014). Corroborating this assumption, foliar phosphate levels were explicitly depleted upon selenite exposure, while selenate had no impact, suggesting direct competition between selenite and phosphate uptake. Phosphate uptake and translocation is achieved by two membrane resident phosphate transporters, PHT1 and PHO1 (Młodzińska and Zboińska, 2016; Wege et al., 2016). PHT1 consists of 12 transmembrane domains and acts as a proton anion symporter in the plasma membrane, capable of transporting selenite (Zhang et al., 2014; Gu et al., 2016). Several PHT1 isoforms contribute to phosphate uptake from the soil. In contrast to PHT1, PHO1 is not required for phosphate uptake into root cells but is essential for loading the xylem with phosphate. Since PHO1 had no intrinsic transporter activity when expressed in yeast or Xenopus oocytes, it was postulated to act as a gatekeeper of phosphate xylem loading by regulating PTH1 activity (Hamburger et al., 2002). Consequently, *pho1* mutants suffer from a shoot phosphate deficiency, albeit root phosphate uptake is unaffected (Poirier et al., 1991; Stefanovic et al., 2007). It is conceivable to hypothesize that this regulatory function of PHO1 for xylem loading *via* PHT1 contributes to trapping selenite into the roots, albeit selenite uptake by PHT1 isoforms is not affected.

In contrast to selenite, selenate is taken up by the well-characterized sulfate transporter system, with SULTR1;2 being the primary importer of selenate and sulfate from the soil (Hawkesford et al., 1993; El Kassis et al., 2007; Rouached et al., 2008). Since sulfate directly competes with selenate for uptake in non-Se-hyperaccumulators of the *Brassicaceae* family (El Mehdawi et al., 2018), the substantial accumulation of foliar sulfur upon selenate application must be caused by upregulating the sulfate translocation pathway (Figure 2C; Hsu et al. (2011)). In support of this view, transcriptional induction of the assimilatory sulfate reduction pathway has been previously shown upon selenate treatment in Arabidopsis (Van Hoewyk et al., 2008). Furthermore, short-term selenate application triggered significant sulfate translocation from the root to the shoot (Schiavon et al., 2012). Consistently with these reports, we found enhanced total sulfur and sulfate levels in the leaves of selenate-treated Arabidopsis plants. In addition, low-molecular-weight thiols and OAS accumulated in leaves of the wild type upon selenate treatment. A similar pattern of metabolic adaptation (growth retardation in combination with accumulation of sulfate, thiols and OAS) also occurred in the *sir1-1* mutant, displaying only 30% of wild-type sulfite reductase activity in leaves (Khan et al., 2010). The substantial overlap in the metabolite response of selenate treated wild type and the *sir1-1* mutant allows us to hypothesize that selenate reduction partially outcompeted sulfate reduction in Arabidopsis plants treated with 50 μM selenate. A hallmark of decreased sulfate reduction in leaves is the accumulation of OAS, which can trigger transcription of high-affinity sulfate transporter 1;1 in roots [reviewed in Takahashi et al. (2011)]. Also, in *sir1-1*, the sulfur-starvation signal OAS accumulated in leaves. Furthermore, the grafting of the *sir1-1* scion to wild-type rootstock triggered foliar sulfur accumulation, demonstrating a dominant role of the shoot over the root with respect to sulfur-related gene expression, root sulfate uptake, and organic sulfur metabolites (Forieri et al., 2021). Selenite accumulation in roots did not trigger sulfate uptake, even though the root is the primary organ for sulfur deficiency perception (Buchner et al., 2004; Durenkamp and De Kok, 2004; Hubberten et al., 2012) and selenite caused accumulation of thiols and OAS in roots (Figures 3B–D). Most likely, the inhibitory impact of the thiols cysteine and glutathione prevented significant induction of sulfate transporters by root OAS accumulation (Koprivova et al., 2000; Hesse et al., 2003). In contrast to selenite application, thiol levels in roots were not affected upon selenate application (Figures 3B,C), because selenate was predominantly transported to the shoot for reduction while selenite was trapped in roots (Figure 2).

On top of the discriminative transport of Se species, this study uncovers Se-induced oxidation of the glutathione pool as a likely trigger of Se toxicity. The most striking difference between Se toxicity induced by selenate or selenite is the chlorotic phenotype of selenate-treated plants (Figure 1C). Only in selenate-treated plants, the plastidic glutathione pool in leaves was significantly oxidized when compared to control, strongly suggesting oxidative stress in plastids (Figures 6B,D). Since oxidative stress and perturbation of the plastidic glutathione production and redox homeostasis are known triggers of chlorosis in diverse plant species (Creissen et al., 1999; Gomez et al., 2004; Herschbach et al., 2010), the observed chlorotic phenotype of selenate-treated Arabidopsis plants might be explained by the plastidic redox impairment. The oxidized cytosolic glutathione pool (Figures 6A,C) is prone to contribute to the slow growth phenotype of selenate-treated plants since oxidation of the cytosol causes transcriptional induction of diverse defense pathways [summarized in Huang et al. (2019)]. Indeed, many defense-related genes were significantly induced upon selenate treatment in leaves and roots of Arabidopsis (Van Hoewyk et al., 2008). However, the trade-off between growth and defense to selenate-induced growth retardation needs further validation.

Selenite-induced ROS formation has been shown previously in plant cells (Lehotai et al., 2012) and in other eukaryotic cells was proposed to be caused by selenite-induced glutathione-persulfide production resulting in superoxide formation (Spallholz, 1994; Wallenberg et al., 2010). In contrast to selenite, selenate-induced impairment of the glutathione redox milieu was not known prior to this study. How selenate triggered glutathione oxidation in plants remains unknown, but the *in vitro* analyses (Figure 4C) rule out direct oxidation of GSH by selenate. Instead, the time-resolved comparison of selenite-induced and selenate-induced GSH oxidation strongly suggested that both Se species must be further reduced and incorporated into Se-Cys in the plastids to mediate an impact on glutathione oxidation (Figure 5). These findings support the hypothesis that misincorporation of Se-Cys into proteins rather than selenate or selenite causes ROS formation (Van Hoewyk, 2013). Prime candidates for inactive Se-Cys containing proteins are redox-active Fe/S-cluster containing proteins located in the plastidic electron transport chain, e.g., photosystem II, whose malfunction leads to electron spillover causing superoxide formation and/or false sensing of ROS signals (Foyer and Noctor, 2003; Derks et al., 2015).

Our study shed light on the oxidation state-specific differences of selenium toxicity in the model dicotyledonous plant *A. thaliana*. These novel findings may help to select the best Se species for fertilization of crops *via* the pedosphere. Our findings suggest that selenate is superior to selenite for pedospheric Se fertilization of dicotyledonous crops due to its better mobility in Arabidopsis. Further studies are required to understand how selenate and selenite cause subcellular-specific oxidation of the glutathione pool and if this oxidation contributes to the Se-induced slower growth of Arabidopsis.

## Data Availability Statement

The original contributions presented in the study are included in the article/Supplementary Material; further inquiries can be directed to the corresponding author.

## Author Contributions

MK performed the elemental analysis (Figure 2), the initial selenium feeding experiments, and helped IW and MP with plants growth and harvesting (Figure 2). IW and MP grew plants on solidified agar medium plates and in hydroponic culture and assisted in analyzing metabolites (Figures 1–3). AS performed the *in vitro* assays [Figure 4 and *in vivo* Grx1-roGFP2 quantification (Figures 5, 6)] under supervision of AM. MW supervised the quantification of metabolites shown in (Figure 3). TR and RH designed the study. MW, MK, and RH wrote the manuscript. All authors contributed to the article and approved the submitted version.

## Funding

Research at Heidelberg was funded by the German Research Council (DFG) *via* WI 3560/1–2 and WI 3560/2–1 to MW and HE 1848/15–2 and HE 1848/16–1 to RH.

## Conflict of Interest

The authors declare that the research was conducted in the absence of any commercial or financial relationships that could be construed as a potential conflict of interest.

## Publisher’s Note

All claims expressed in this article are solely those of the authors and do not necessarily represent those of their affiliated organizations, or those of the publisher, the editors and the reviewers. Any product that may be evaluated in this article, or claim that may be made by its manufacturer, is not guaranteed or endorsed by the publisher.
